# Cancer Metastasis as Disrupted Developmental Phenotype

**DOI:** 10.2174/138920208783884919

**Published:** 2008-03

**Authors:** Hideshi Ishii, Toshiyuki Saito

**Affiliations:** 1 Center for Molecular Medicine, Jichi Medical University, Tochigi; 2 National Institute of Radiological Science, Chiba, Japan

**Keywords:** Cancer, metastasis, notch, Bmp, SHH.

## Abstract

Cancer metastasis is a complex processes, associated with the invasion to tissues with extensive degradation of the surrounding normal components, penetration into vessels, circulation, and then invasion to normal tissues in body. It would be not surprising that tumor cells usurp pathways critical to the developing embryo during metastasis. For the better understanding of tumor metastasis, this review will highlight the recent progress and significance of the signal transduction pathways, relevant to developmental biology.

## INTRODUCTION

Cancer deaths result from the local invasion and distant metastasis of tumor cells in ~90% of cases [1]. Cancer metastasis is a complex series of processes, fundamentally associated with the invasion, which is the direct migration and penetration by cancer cells into neighboring tissues. Cancer cells leave the original tumor site, migrate and invade to tissues with extensive degradation of the surrounding normal components such as extracellular matrix, penetrate into lymphatic and blood vessels, circulate through the bloodstream, and then invade normal tissues elsewhere in the body. During the process of primary and metastasized sites, one of critical events required is the growth of a new network of blood vessels, i.e., angiogenesis [2]. For our better understanding of their roles in regulating tumor metastasis, this review will highlight the significance of signal transduction pathways in developmental biology. It would be not surprising that tumor cells usurp pathways critical to the developing embryo, as many of the normal developmental programs include processes that are also seen during tumor metastasis, including epithelial to mesenchymal transition (EMT), tissue specific morphogenesis, cellular motility and invasion [3].

## DEVELOPMENTAL SIGNAL PATHWAY IN CANCER

The observation reinforces that the aggressive tumor cells that metastasize from the primary sites often usurp pathways that function during normal development [3]. Recent studies indicate that embryonic pathways have been shown to affect the survival of tumor initiating or stem cells and to orchestrate a complex microenvironment, i.e., niche, that promotes tumor survival and progression [3]. Increased motility and invasiveness of certain cancer cells is associated with the process, the epithelial-to-mesenchymal transition (EMT), which occurs during embryonic development [4]. EMT is an important change in cell phenotype, which allows the escape of epithelial cells from the structural constraints imposed by tissue architecture [5]. Although the observations of morphology of various tumors indicated epithelial and mesenchymal components of tumors as metaplasia [6, 7], the EMT phenomenon was recognized by Elizabeth Hay in the early to mid1980’s to be a central process in early embryonic morphogenesis [4] and the phrase of epithelial to mesenchymal transition was appear in 1980’s with reference to the study of a cellular change elicited by extracellular matrix [8], and this phenomenon was further characterized by Hay [4]. 

Recent studies indicate that this transition phenomenon is the result of a complex physiologic process that includes dissolution of adherens junctions, loss of cell polarity, a change to spindle-like cell morphology, increased cell motility, loss of epithelial markers, and induction of mesenchymal markers [9-11]. The expression of vimentin is increased and E-cadherin mediated cell adhesion is perturbed in this process [10, 12]. Over the last decade, evidence has mounted for cancer genetics, that they are fundamentally diseases, which associate genetic and epigenetic alterations, requiring the accumulation of genomic alterations that inactivate tumor suppressors and activate proto-oncogenes [13, 14]. The accumulation occurs in a stepwise manner, which progress from very early stage with a few or several alterations in human genome to advanced stages with numerous alterations leading to aggressive characteristics. Classical tumor suppressors such as retinoblastoma 1 and p53 and oncogenes such as *RAS* and *MYC* have been extensively studied [15, 16]. But recent studies also implicate numerous alterations, such as alterations of non-protein-coding RNAs expression and DNA damage checkpoint responses, are associated with structural aberrations of tumor suppressor and oncogene loci [17]. Those are involved in complicated and overlapping pathways that regulate the biologically important processes including cell-cycle progression, gene expression, DNA damage response and apoptosis [18].

The multi-step model of tumor progression emphasizes the accumulation of genetic alterations as the central mechanism driving tumorigenesis [13, 14]. In this view, it is indicated that the normal cell is an almost passive recipient of the mutations, and its cancer-associated phenotypes are governed largely by somatic mutations during the course of tumor progression [19, 20]. While the role of somatic mutations has been extensively documented in determining tumor phenotype and many of the observed differences have been explained among different tumors [20], the biological regulation of carcinogenesis and development of tumors in the microenvironment has been extensively studied in developmental biology and our emerging understanding of their roles in regulating tumor metastasis in the view of critical signal transduction pathways: notch, bone morphogenic protein (Bmp) and sonic hedgehog (Shh).

## EACH SIGNAL TRANSDUCTION 

### 1 Notch

Notch signaling is critical for cell-cell communication and regulates a broad spectrum of cell fate decisions during embryonic development and in the adult organism *via *stem cell proliferation, differentiation and cell death [21]. Notch is instrumental in regulating processes such as neurogenesis, somitogenesis and angiogenesis [22]. Notch proteins are members of the conserved transmembrane receptor family including four members, Notch 1 to 4 [21]. The Notch genes encode transmembrane receptors, which contain a large extra-cellular domain, composed of a variable number of epidermal growth factor (EGF)-like repeats and an intracellular signaling domain, which consists of six ankyrin/cdc10 motifs and nuclear localization signals [23]. Notch receptors interact through their extracellular domain with other membrane-associated ligands, the Delta and Serrate/ Jagged families, which are composed of five proteins, Jagged1 and 2 and Delta-like 1, 3 and 4 [23]. Notch signaling is activated by ligand-receptor interaction and triggers proteolytic cleavages by the γ-secretase complex, which releases the Notch intracellular domain into the nucleus. Notch intracellular domain binds to the CBF1 DNA binding protein of the transcriptional activator complex, the activation of which can lead to the expression of target genes, such as Hes family genes, involved in cell growth and differentiation. 

A progenitor cell with induced levels of Delta by Notch signaling during neuronal differentiation becomes a neuron and sends inhibitory signals to other progenitor cells to maintain their undifferentiated status, which inhibits the expression of Delta [24]. But Notch signaling is shown also to promote angiogenesis and EMT [25]. Notch1 and Notch4, along with Delta4 and Jagged1 play a role in the development of cardiac and vascular systems during embryonic development through downstream effectors of Notch signaling and target genes such as *RBPJk*, *HRT1*, and *HRT2* [25]. Notch1 and Notch4 double deficient mice have apparently normal vasculogenesis, but they show impaired angiogenesis in the embryo proper and placenta [26]. The study of Notch1 mutants have a collapsed endocardium and show an absence of mesenchymal cells in the cardiac cushions, indicating that Notch has a significant role during cardiac development in the process of EMT [25]. Collectively, Notch is a critical mediator of both angiogenesis and EMT.

Alterations of the Notch signaling pathway have been implicated in cancer [3]. Recent studies indicate significant involvement of the Notch signaling pathway in initiation and development of breast cancer (reviewed in [27-29]). The oncogenic function of Notch1 and 4 is shown by studies of the mammary epithelial cell system [27-29]. Transgenic overexpression of the Notch intracellular domain of Notch1 and Notch3 resulted in the development of mammary tumors [30]. However, demonstrating a linkage of cancer development or metastasis with EMT, the potentially rapid and transient process *in vivo* has proven difficult and data connecting the relevance of this process to tumor progression is still somewhat limited and controversial. Indeed Notch signaling has been classified as either oncogenic or tumor-suppressive depending on the cell type, specific type of mutation within the Notch pathway, the timing in the context of transformation and metastasis and the tissue context [3, 31]. 

Stephen Paget’s 1889 proposal [32] that metastasis depends on cross-talk between selected cancer cells (the ‘seeds’) and specific organ microenvironments (the ‘soil’) still holds forth today [33], indicating the notion that the potential of a tumor cell to metastasize depends on its interactions with the homeostatic factors that promote tumor cell growth, survival, angiogenesis, invasion and metastasis [33]. A regulatory mechanism enabling certain tumor stem cells from a primary site to survive in further spreading is depending on the tissue of origin and the route of spread of metastasis, and is highly consistent with the seed and soil hypothesis [3, 33]. Considering Notch signaling is regulated by timing and signal strength, the number of ligand-receptor system expressed on a tumor stem cell will directly affect niche interactions, and the biological effect is codependent on maintenance of survival and avoidance of induction of apoptosis in cancer-initiating cells with carrying deleterious damages, and suggests that targeted suppression of the survival signaling pathway may give the rationale for sensitizing cancer-initiating cells to novel therapeutic approach.

### 2 Bmp

Bmps, members of the Tgf-ß family of signaling proteins, are secreted ligands that signal *via *autocrine and paracrine mechanisms to regulate cell proliferation and differentiation [34, 35]. Bmp ligands bind to cell surface-associated proteins called bone morphogenic receptors type I and type II [34, 35]. Bmp proteins have also been associated with EMT. In cardiac cushion development, Bmp2 has been associated with EMT [36]. Bmp2 was shown to enhance formation of the cardiac jelly, to pattern the atrioventricular myocardium and to induce endocardial EMT [36]. Bmp2 is required for myocardial expression of Has2, a crucial component of the cardiac jelly matrix [36]. During EMT, Bmp2 promotes expression of the basic helix-loop-helix factor Twist1, implicated in EMT in cancer metastases, and the homeobox genes Msx1 and Msx2, indicating that Bmp2 has a crucial role in coordinating multiple aspects of AV canal morphogenesis [36]. On the other hand, Bmp4 has been shown to induce EMT in human ovarian cancer cells [37]. Using primary human normal ovarian surface epithelial and epithelial ovarian cancer cells, it was shown that treatment with Bmp4 increased cellular adhesion, motility and invasion with up-regulation of EMT markers Snail and Slug and with down-regulation of E-cadherin, which are closely relevant to changes in the level of activated Rho GTPases [37]. A link between autocrine Bmp signaling mediated through the Rho GTPase family and Snail- and Slug-induced EMT is proposed and suggested that it may collectively contribute to aggressive ovarian cancer behavior [37]. Bmp7, however, has been shown to antagonize Tgf-induced EMT [38-41], in the representative study of renal cells and in renal cell injury by systemic administration of recombinant human Bmp7, which led to repair of severely damaged renal tubular epithelial cells, in association with reversal of chronic renal injury by counteracting to Tgf-β1-induced EMT and reverses chronic renal injury, indicating evidence of cross talk between Bmp7 and Tgf-β1 in the regulation of EMT [41]. As indicated in organogenesis, ‘mesenchymal’ cells may possess remarkable plasticity and can regain a fully differentiated epithelial phenotype *via *a mesenchymal-to-epithelial transition (MET) [38-40], Bmp7 is known to induce MET in normal and nontransformed cells [42, 43]. A cross-talk between Bmp7 and Tgf-β  signaling in the regulation of EMT in breast cancer is proposed, and Bmp7 was identified as a potential key molecule in therapy for metastatic bone disease [11].

### 3 SHH

The hedgehog family of signaling proteins, hedgehog ligands, Sonic (Shh), Indian (Ihh), and Desert (Dhh), are secreted proteins, which signal through autocrine and paracrine mechanisms to control cell proliferation, differentiation, and morphology [44]. The hedgehog proteins exert their function by binding to a 12-pass transmembrane protein called Patched (Ptch) [45], which relieves the inhibitory affect of Ptch on a serpentine protein called Smoothened (Smo), leading to hyper-phosphorylation of SMO [46, 47]. The signal pathway induces the activation and repression of target genes through the Gli family of transcription factors, Gli-1, -2, -3, which regulate the transcription of target genes.

SHH is shown to affect EMT [4, 48]. Disruption of Shh signaling by the inhibitor cyclopamine inhibited EMT in pancreatic cancer cell lines [48]. In the study, Shh inhibition with cyclopamine resulted in down-regulation of snail and up-regulation of E-cadherin, consistent with inhibition of EMT, and was mirrored by a striking reduction of *in vitro* invasive capacity, indicating that blockade of Shh signaling inhibits pancreatic cancer invasion and metastases [48]. It is suggested that by targeting specific cellular subpopulations, such as cancer-initiating cells or cancer stem cells likely involved in tumor initiation at metastatic sites, Hh family inhibitors may provide a new paradigm for therapy of disseminated malignancies [48]. The stem cell targeting may be moiré realistic, particularly when used in combination with conventional anti-metabolites, which can reduce "bulk" tumor size, and eliminate so-called metastatic cancer stem cells [48].
